# Inference of marker genes of subtle cell state changes via iLR: iterative logistic regression

**DOI:** 10.1093/bioinformatics/btag051

**Published:** 2026-02-02

**Authors:** Yingtong Liu, Aaron G Baugh, Evanthia T Roussos Torres, Adam L MacLean

**Affiliations:** Department of Quantitative and Computational Biology, Dornsife College of Letters, Arts and Sciences, University of Southern California, Los Angeles, CA 90089, United States; Division of Medical Oncology, Department of Medicine, Keck School of Medicine, Norris Comprehensive Cancer Center, University of Southern California, Los Angeles, CA 90033, United States; Division of Medical Oncology, Department of Medicine, Keck School of Medicine, Norris Comprehensive Cancer Center, University of Southern California, Los Angeles, CA 90033, United States; Department of Quantitative and Computational Biology, Dornsife College of Letters, Arts and Sciences, University of Southern California, Los Angeles, CA 90089, United States

## Abstract

**Motivation:**

Differential expression and marker gene selection methods for single-cell RNA-sequencing (scRNA-seq) data can struggle to identify small sets of informative genes, especially for subtle differences between cell states, as can be induced by disease or treatment.

**Results:**

We present iterative logistic regression (iLR) for the identification of small sets of informative marker genes. iLR applied logistic regression iteratively with a Pareto front optimization to balance gene set size with classification performance. Benchmarking iLR on *in silico* datasets, we demonstrated its comparable performance to the state-of-the-art at single-cell classification using only a fraction of the genes. We then tested iLR on its ability to distinguish neuronal cell subtypes in healthy versus autism spectrum disorder patients and find that it achieves high accuracy with small sets of disease-relevant genes. Applying iLR to investigate immunotherapeutic effects in cell types from different tumor microenvironments, we found that iLR infers informative genes that translate across organs and even species (mouse-to-human) comparison. We predicted via iLR that entinostat acts in part through the modulation of myeloid cell differentiation routes in the lung microenvironment. Overall, iLR provides means to infer interpretable transcriptional signatures from complex datasets with prognostic or therapeutic potential.

**Availability and implementation:**

iLR is freely available at GitHub https://github.com/maclean-lab/iLR and Zenodo https://zenodo.org/records/17728797.

## 1 Introduction

Single-cell resolution has proven essential for resolving cell states and their corresponding gene expression differences in biological systems. Single-cell RNA-sequencing (scRNA-seq) quantifies gene expression at the single cell level, and with decreasing costs and a proliferation of protocols, has been widely adopted (Svensson *et al.* 2020). Downstream of cell clustering to identify cell states is the identification of marker genes that distinguish cells by their type, state, condition, etc. (Huang and Zhang 2021, Pullin and McCarthy 2024). Marker genes are critical for interpreting cell clusters as different cell states. They are also essential for comparative analyses of cells across treatments, genetic perturbations, or disease states (Dawson and Kouzarides 2012, Falkenberg and Johnstone 2014, Perri *et al.* 2017). When such comparisons reveal subtle transcriptional differences between groups, they can be difficult to capture with conventional marker gene selection approaches. For example, some epigenetic therapies induce broad shifts in gene expression without affecting target genes directly (Falkenberg and Johnstone 2014). Even though certain epigenetic modulators have been approved and are being used as cancer therapeutics, their precise mechanisms of action often remain unclear (Dawson and Kouzarides 2012, Perri *et al.* 2017). Furthermore, combination therapies—designed to enhance efficacy and prevent drug resistance—are increasingly common in clinical practice (Fitzgerald *et al.* 2006). These regimens often produce complex and context-dependent transcriptional responses, making it essential to identify condition-informative gene sets to elucidate therapeutic mechanisms and optimize treatment strategies.

A key challenge in the analysis of single-cell datasets to perturbations due to, e.g., cancer or developmental disorders (Chea *et al.* 2023) is the identification of informative gene sets that capture the (often widespread but subtle) transcriptional differences that occur. Canonical statistical methods for differential expression testing, such as DESeq2 (Anders and Huber 2010), edgeR (Robinson *et al.* 2010), or the Student’s *t* or Wilcoxon rank-sum tests, are commonly applied (Wilcoxon 1945). These approaches rely on ranking genes by statistical significance, and it can be difficult to select compact, interpretable gene sets—particularly when changes are small in magnitude but biologically meaningful.

A number of methods have been developed specifically for marker gene selection in the context of cluster-based cell state identification (Vargo and Gilbert 2020, Guan *et al.* 2021, Dumitrascu *et al.* 2021, Nelson *et al.* 2022, Sun and Qiu 2024) or label-free (Wang *et al.* 2025). These approaches are effective at identifying genes that distinguish well-separated clusters, thereby facilitating cell type or state annotation (Pullin and McCarthy 2024), but they are not always well-suited for detecting markers when the perturbation- or treatment-induced expression changes are subtle, widespread, and may not be tightly aligned with cell state boundaries. Moreover, few methods provide principled means to control the size and the redundancy of the gene set, which can be critical for interpretability and downstream validation. Logistic regression is a simple and interpretable method for classification (Berkson 1944, Cox 1958). The coefficients of a fitted logistic regression model provide means for ranking features by importance, enabling feature selection. Logistic regression has demonstrated competitive performance in scRNA-seq classification (Huang and Zhang 2021, Le *et al.* 2022) and marker gene selection (Pullin and McCarthy 2024).

We developed iterative logistic regression (iLR) to combine cell classification and feature selection by iteratively refining a set of features via logistic regression. We used Pareto front optimization to balance the gene set size with classification accuracy, enabling the inference of small, informative gene sets. iLR is designed to capture condition- or treatment-driven differences, providing interpretable signatures while maintaining high classification accuracy—even for weak signals with high biological variability.

The remainder of this paper is organized as follows. In the next section, we discuss the methods and implementation of iLR. We then assess the performance of iLR on *in silico* datasets against standard and state-of-the-art feature selection and classification methods. To test iLR on real datasets, we evaluated iLR on a large human scRNA-seq dataset comprising multiple neuronal samples from autism patients and healthy controls (Velmeshev *et al.* 2019) and demonstrated that iLR can accurately distinguish differences with small sets of informative genes. Finally, we applied iLR to immune cell types in breast and lung metastatic tumor microenvironments (TMEs) to identify genes that mark for changes due to combination therapy (Sidiropoulos *et al.* 2022, Roussos Torres *et al.* 2024). In combination with downstream analysis via gene regulatory network inference, we identified putative mechanisms of action of combination therapy in immune cell subtypes.

## 2 Materials and methods

### 2.1 Overview of iLR

The goal of iLR is to predict small sets of genes that are informative of biological processes underlying differences across conditions in single-cell RNA-sequencing (scRNA-seq) data. iLR uses a logistic regression model for classification, i.e. to identify features (genes) that can accurately classify single cells into binary groups, and obtains feature importance scores from the logistic regression coefficients.

**Figure 1:**
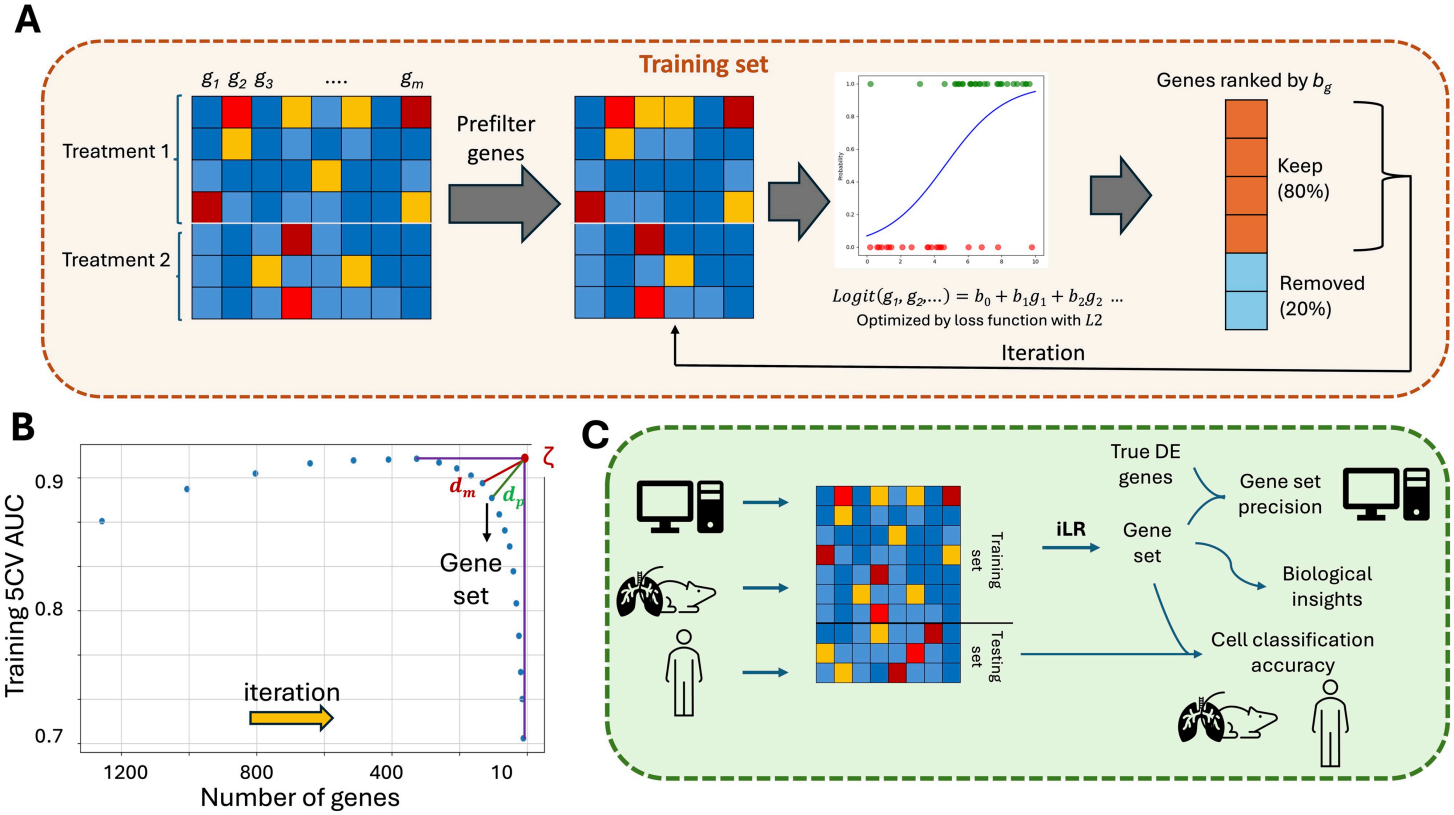
Overview of iLR. (A) The pipeline employed by iLR to select genes based on iterative logistic regression. (B) A Pareto front approach is taken to select the optimal gene set. Each point indicates one gene set: over successive iterations, the gene set size decreases. ζ marks the point of maximum AUC and minimum gene number from which the Pareto optimum is found via $d_{m}$, the point that minimizes the distance to ζ among possible gene sets. In cases where a penalty is applied, $d_{m}$ is replaced with $d_{p}$, the penalized Pareto optimal gene set. (C) Training, testing, and applications of iLR are performed on simulated, murine, and human scRNA-seq datasets. Gene sets are evaluated against the ground truth, the literature, or comparatively across datasets from different organs or species.

As input, scRNA-seq datasets are split into training and testing sets for each evaluation. For a given dataset, a logistic regression classifier is applied to the training set given by *n* cells and *m* genes in an iterative manner. At each iteration, a logistic regression model is fitted:


$$\text{logit}(g_{1},g_{2},\ldots ,g_{m})=b_{0}+b_{1}g_{1}+b_{2}g_{2}+\cdots +b_{m}g_{m},$$

regularized by an L2 penalty with regularization strength set by *C*. Each coefficient $b_{g}$ (for gene *g*) is used as a measure of the importance of *g* in the logistic regression classifier. Genes are ranked by the coefficient strength, and the top 80% genes are kept for the next iteration of logistic regression (Fig. 1A). The algorithm proceeds until fewer than 10 genes are left. Five-fold cross-validation accuracy on training, area under the curve (AUC), and the gene set size are recorded at each iteration. iLR was implemented in Python and LogisticRegression with the liblinear solver from scikit-learn (Pedregosa *et al.* 2011).

iLR uses L2 regularization, with a default regularization strength C=0.1. iLR is provided with *C* as a parameter that can be set by the user. For most uses, we recommend C∈[0.1,0.01]. For comparison of regularization (L1 versus L2, each with *C* = 1, $10^{-1}$, $10^{-2}$, $10^{-3}$, $10^{-4}$, and $10^{-5}$), we defined an F1-like score defined as


$$\frac{2*\text{precision}*\text{AUC}}{\text{precision}+\text{AUC}},$$

which summarizes five-fold validation training classification accuracy (AUC) and gene set precisions through a single score. We refer to Section 3.1 for a discussion of the impact of regularization strength on the iLR results.

### 2.2 Gene set size selection by Pareto front optimization

We propose that optimal gene sets should maximize classification performance (AUC) and minimize gene set size, since small gene sets are most actionable. To achieve this, we use a Pareto front-based analysis: the Pareto front provides a means to perform multiobjective optimization. Given the goal of finding a gene set with a small size and high classification AUC, Pareto from optimization proceeds as follows. Normalize AUC and gene set size to [0,1] and find the intersection point (ζ) between the highest AUC and the lowest gene set size (Fig. 1B). The optimal point (the Pareto front) is that which minimizes the distance $d_{m}$ to ζ (Fig. 1B). We extend the approach using a Pareto front penalty (ϵ), which reduces the gene set size at the expense of classification accuracy. The reasoning behind such a penalty is that relatively small reductions in the AUC for relatively large reductions in the gene set size are desirable. We apply a penalty ϵ to increase the search area around the Pareto front. That is, the penalized gene set is defined as the smallest gene set within a distance of $\left(d_{m}+\epsilon \right)$ around ζ; the distance that defines the penalized gene set is then $d_{p}>d_{m}$ from ζ (Fig. 1B). To infer robust gene sets with iLR, 10 rounds of Pareto front optimized iLR are performed, and those genes that appear more than 5 times in total are included in the final gene set.

### 2.3 Simulating scRNA sequencing data with Splatter

**Figure 2:**
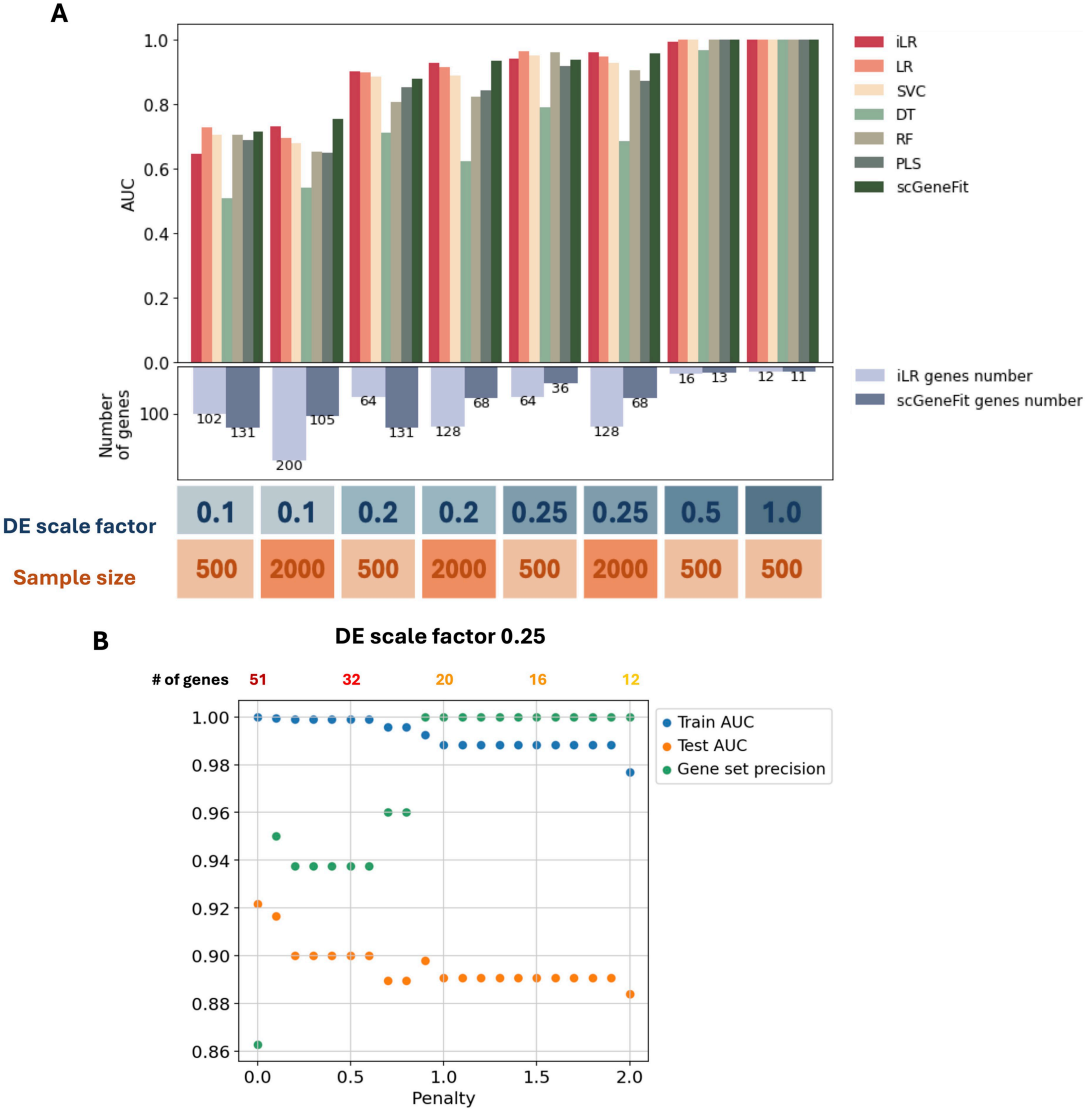
Benchmarking iLR on simulated datasets. (A) Comparison of iLR with other methods for classification via the AUC on simulated scRNA-seq datasets with different DE scale factors and sample sizes. The number of genes applies only to iLR and scGeneFit combined with Pareto front without penalty. (B) The impact of the Pareto front penalty at scale factor 0.25 on the training & test AUC and the gene set precision. DT, decision tree; PLS, partial least squares; RF, random forest; SVC, support vector classifier.

To evaluate iLR against data for which we know the ground truth, we used the R package Splatter (Zappia *et al.* 2017) to simulate scRNA-seq data. While there exist significant challenges in the use of simulated scRNA-seq data for benchmarking (Crowell *et al.* 2023), it remains a useful tool in combination with other analyses. We used Splatter to estimate the parameters needed to simulate a synthetic dataset based on input from a real scRNA-seq dataset, including the biological coefficient of variation, the dropout distribution, and the library distribution (Zappia *et al.* 2017). Via Splatter, we can then simulate different scenarios by changing the extent to which simulated genes are differentially expressed (DE). As an input sample, we used granulocytic myeloid-derived suppressor cells (G-MDSCs) from untreated murine lung metastases, with 1202 genes input to Splatter that were selected by logistic regression with L1. We varied two Splatter parameters to generate a total of eight datasets: the number of cells in the sample and the DEscalefactor, which defines the extent of the log fold changes in DE genes. The DEscalefactor was varied from 0.1 to 1 to capture very small to very large log fold change differences in gene expression (Fig. 2A). The fraction of DE genes in each simulated dataset was held fixed at 0.1. The sample size was tested by varying the number of cells from either 500 or 2000. Each simulated dataset was balanced, i.e. 50% of cells in each condition (control and perturbed). To test the impact of data imbalance on iLR performance, we simulated six additional datasets, consisting of either 500 or 2000 cells with slight (4:6), moderate (2:8), and extreme (1:9) imbalances between the two conditions at a DE scale factor of 0.25. The precision of the inferred gene set was calculated based on the known DE genes, and was used as a metric to assess the performance of iLR (Fig. 1C). Splatter-generated raw count matrices were normalized to $10^{4}$ counts per cell and then log-transformed. Training and test datasets were splitted at the ratio 8:2.

### 2.4 Benchmarking iLR against alternative classification and marker gene selection methods

To evaluate the capability of iLR in classification, several basic machine learning classification algorithms were applied to the Splatter simulated scRNA-seq data. The following algorithms were compared in performance to iLR (all implemented in scikit-learn). Simple logistic regression was implemented, regularized by an L2 penalty with a rate of 0.1. A linear support vector classification was implemented with a hinge loss. A decision tree was implemented with min_sample_split 10. A random forest was implemented with default parameters, and a partial least squares regression model was implemented with 12 components.

To assess the ability of iLR to accurately identify DE genes, we compared iLR genes to genes identified using a Wilcoxon rank-sum test or scGeneFit (Dumitrascu *et al.* 2021) on simulated scRNA-seq data. scGeneFit utilizes the idea of compressive classification and the largest margin nearest neighbor algorithm to select marker genes for cell states (Dumitrascu *et al.* 2021). To make them comparable, we fixed the gene set size to match the size we got from iLR. We selected the top genes in the Wilcoxon rank-sum test. Then we evaluated the gene sets derived from three methods by comparing them with the true DE genes and their capability of classification. Separately, we applied iLR, the Wilcoxon rank-sum test, and scGeneFit to NT2.5-LM scRNA-seq data to find the genes contributing to the treatment effects.

### 2.5 scGeneFit gene set selection

Similar to iLR, we optimized scGeneFit gene sets using a Pareto front approach. In each round of scGeneFit, we set the number of genes to approximately 80% of the previous round to fairly compare similarly sized gene sets. We also applied a Pareto front approach to the results of multiple runs of scGeneFit, balancing classification AUC and gene set size to infer the optimal number of genes to retain.

### 2.6 scRNA-seq data processing and analysis

#### 2.6.1 snRNA-seq from ASD patients and controls

Neuronal single-nuclei RNA-sequencing (snRNA-seq) data from autism spectrum disorder (ASD) patients and controls were analyzed (Velmeshev *et al.* 2019). Pre-processed data with cluster annotations are available from the UCSC Cell Browser at https://autism.cells.ucsc.edu. This dataset contains 104 559 nuclei from 41 tissues. Total 31 samples with 15 from ASD patients and 16 from controls. Seventeen cell types were identified. Cells expressing <200 genes or more than 8000 genes or having more than 15% mitochondrial gene expression were removed, resulting in a total number of 103 370 cells and 37 762 expressed transcripts.

#### 2.6.2 Lung metastasis scRNA-seq library preparation and sequencing

Primary tumor scRNA sequencing data derived from mice injected with the NT2.5 cell line and treated with vehicle (V), entinostat (E), entinostat with aCTLA4 and aPD1 (EPC), entinostat with aCTLA4 (EC), and entinostat with aPD1 (EP). For library preparation, 10× Genomics Chromium Single-Cell 3′ RNA-seq kits v2 were used. Gene expression libraries were prepared according to the manufacturer’s protocol. RNA was extracted from 20 whole tumors from the following groups: vehicle control (V), entinostat-treated (E), entinostat and anti-PD-1 (EP), entinostat and anti-CTLA-4 (EC), entinostat with anti-PD-1 and anti-CTLA-4 (EPC), with four biological replicates from each of the five experimental groups. Tumors were sequenced in four batches: RunA (eight tumors; 1 E, 2 EP, 2 EC, 2 EPC, 1 V), RunB (eight tumors; 1 E, 2 EP, 2 EC, 2 EPC, 1 V), Pilot1 (two tumors, 1 E and 1 V), and Pilot2 (two tumors, 1 E and 1 V). Each batch had an approximately equal assortment of samples from each treatment group to reduce technical biases. Illumina HiSeqX Ten or NovaSeq were used to generate approximately 6.5 billion total reads.

#### 2.6.3 Alignment and data preprocessing

Paired-end reads were processed using CellRanger v3.0.2 and mapped to the mm10 transcriptome v1.2.0 by 10× Genomics with default settings. ScanPy v1.9.1 (Wolf *et al.* 2018) and Python v3 were used for quality control and basic filtering. For gene filtering, all genes expressed in <3 cells within a tumor were removed. Cells expressing <200 genes or more than 8000 genes or having more than 15% mitochondrial gene expression were also removed. Gene expression was total count normalized to 10 000 reads per cell and log-transformed. Highly variable genes were identified using default ScanPy parameters, and the total counts per cell and the percent mitochondrial genes expressed were regressed out. Finally, gene expression was scaled to unit variance, and values exceeding 10 standard deviations were removed. There were 54 636 cells and 19 606 genes after preprocessing. Batch effects were corrected using the ComBat batch correction package. Neighborhood graphs were constructed using 10 nearest neighbors and 30 principal components. Tumors were clustered together using Louvain clustering and six main clusters were identified (with resolution parameter 0.1).

#### 2.6.4 Application of iLR to lung metastatic and primary tumor samples

We analyzed differences in each TME due to two treatments: entinostat alone (E) or in combination with anti-PD-1 and anti-CTLA4 (EPC) against vehicle (V), because (i) we were most interested in the mechanisms underlying these effects, and (ii) these treatments were shared across two TME datasets (NT2.5 in the primary breast and NT2.5LM in the lunch). This enabled comparison of iLE gene sets across organs. E and EPC are also the most clinically relevant and have been evaluated in clinical trials (Roussos Torres *et al.* 2024).

Given the inherent differences in cell states and gene expression profiles between the two tumor models, we adjusted the normalized count data for cancer cells and mature myeloids. To better isolate genes relevant to treatment effects in cancer cells and avoid confounding by metastatic signatures, we first identified genes that were DE between two metastatic breast cancer cell lines (NT2.5LM and its parental line NT2.5) (Baugh *et al.* 2024). These genes were filtered out prior to applying iLR. We used classification AUC from lung metastasis-trained models tested on primary tumors to determine the optimal number of genes to exclude for each treatment comparison. Specifically, the top 1000 genes were removed in the V versus E comparison, and the top 500 in the V versus EPC comparison. For mature myeloid cells, dendritic cells were excluded from the lung metastasis samples. For each cell type, we applied rank_gene_groups with the Wilcoxon rank-sum test to identify significantly DE genes to be used as input for iLR.

#### 2.6.5 Training dataset composition

For a given cell type undergoing marker gene selection with iLR, train/test splits are composed as follows. A Wilcoxon rank-sum test was used to perform prefiltering, retaining the set of genes that were significant at an adjusted *P*-value threshold of 0.05. This dataset is then split into training and testing sets at a ratio of 7:3 using stratified sampling.

### 2.7 Sensitivity and ablation analysis

To evaluate the stability, we performed iLR with a penalty of two on each cell type in the ASD data (Velmeshev *et al.* 2019) and trained the iLR gene logistic regression model with bootstraps 200 times. The rank of coefficients was recorded over the 200 bootstraps. For each of the models, ablation analysis was performed by removing one gene at a time, and the difference in AUC of the reduced model from the full model was calculated. Spearman correlation was done on the median rank and the rank of AUC drop from ablation analysis to evaluate if the coefficients reflect the true importance of each feature in the model. To perform a meta-analysis across all cell types from the ASD dataset, we used Fisher’s z-transformation by statsmodels (Seabold and Perktold, 2010) to transform the correlation coefficients to z-scores and found the confidence interval.

### 2.8 Gene regulatory network inference based on marker gene sets

To investigate genes underlying treatment effects (V vs E and V vs EPC) in breast cancer, we applied SCORPION (Osorio *et al.* 2024) using gene sets identified by iLR and scGeneFit with Pareto front penalty 0. We used TF-target database Dorothea (Garcia-Alonso *et al.* 2019, Badia-i Mompel *et al.* 2022, Müller-Dott *et al.* 2023) and protein–protein interaction database STRING (Szklarczyk *et al.* 2023). SCORPION was run with default parameters. Comparisons included iLR and scGeneFit gene sets under both treatment conditions. SCORPION was run with default parameters, and low-weight edges in the TF-target gene network were filtered to reduce noise. Using the vehicle group as baseline, we removed the bottom 50% of edges by weight, which accounted for only about 12% of the total network weight. The same cutoff was applied to the treatment groups for consistency.

To identify transcription factors (TFs) whose regulatory roles were significantly altered by treatment, we performed Wilcoxon tests separately on activating and repressing interactions. Volcano plots were used to highlight TFs with significant changes in regulatory activity.

SCORPION was applied on iLR gene set with penalty 2 from L2/3 cell type in ASD data (Velmeshev *et al.* 2019). igraph (Csárdi *et al.* 2025) in R was used for topological analysis. For each TF and each target gene, out-edges or in-edges were counted, and the TFs and target genes with top increased/decreased edges across controls and ASD patients were identified. Large GRN was constructed by using the Wilcoxon test to identify DE genes with a *P*-value <0.05.

### 2.9 Statistics

Hypergeometric tests were used to assess the significance of the overlap of two gene lists. iLR gene lists from human ASD and control snRNA sequencing data were evaluated by overlapping with all the genes in the SFARI Gene Module database (Abrahams *et al.* 2013), which contains genes having evidence of genetic association with ASD. A Bonferroni correction was applied to account for multiple testing. Biological processes were analyzed via gene ontology (GO) analysis (Feuermann *et al.* 2025) in the Python package GSEApy (Fang *et al.* 2023).

## 3 Results

### 3.1 iLR classifies single cells accurately with small sets of genes from simulated scRNA-seq data

We developed iLR to infer small sets of informative genes that mark subtle transcriptional differences between groups of cells. The novelty of iLR comes through the coupling of a logistic regression model with Pareto front optimization to enable optimization between model sparsity and accuracy. In doing so, iLR can identify minimal gene sets from scRNA-seq data, shifting the focus from broad predictive modeling to precise target identification.

To test the ability of iLR to classify cells from scRNA-seq data and identify useful gene sets, it was applied to eight scRNA-seq datasets simulated via Splatter (Zappia *et al.* 2017) with different scales of log fold change of gene expression between conditions and different sample sizes. The gene set is selected based on the AUC via the Pareto front (Fig. 1). Overall, we found that iLR outperformed alternative methods for cell classification, especially with relatively small-scale factors (Fig. 2A, Fig. 1, available as supplementary data at *Bioinformatics* online). Moreover, iLR classified cells accurately with many fewer genes (1%–16% of the total genes) than alternative approaches. scGeneFit applied with a Pareto front (as in iLR) performed similarly in terms of classification accuracy across different DE scales (Fig. 2A). At the smallest scale factors (of size 0.1), we found that increasing the sample size from 500 to 2000 improved classification accuracy; here, iLR selected larger sets of genes. Overall, there is a trade-off between larger sample sizes and smaller scale factors required larger gene set sizes to achieve high AUC.

We found that applying a penalty to the Pareto front optimization to favor smaller gene set sizes over the highest AUC allowed iLR to identify smaller gene sets without incurring large reductions in the test AUC while reducing the training AUC, i.e. reducing the risk of model overfitting (Fig. 2B, Fig. 1, available as supplementary data at *Bioinformatics* online).

Notably, at a scale factor of 0.25, applying Pareto front analysis with a penalty ≥0.9 was able to identify a small, informative gene sets containing all the true DE genes.

Wilcoxon rank-sum tests can often identify good candidate sets of marker genes (Pullin and McCarthy 2024), but they are not designed to control for the number of significant genes. As such, applied to Splatter datasets, the number of identified genes at a *P*-value cutoff of .05 varied widely from 4 to 222 (Table 1, available as supplementary data at *Bioinformatics* online): the number of DE genes increases with the sample size and the scale factor. This can be mitigated by incorporating a Pareto front approach with penalty constraints. For instance, with a penalty of 2, the gene set size was limited to around 20 genes (Fig. 1, available as supplementary data at *Bioinformatics* online).

We examined how data imbalance affected iLR performance. Using Splatter to simulate datasets with different proportions of cells per group (at a DE scale factor of 0.25), we found that iLR demonstrated stability when the sample size is large. With sufficiently many cells (*N* = 2000), even at an imbalance ratio of 1:9, the test AUC exceeded 0.92, and the gene set precision (proportion of selected markers that are in the ground truth) remained stable (Table 2, available as supplementary data at *Bioinformatics* online). With small sample sizes (*N* = 500) we did observe that highly skewed groups compromised the gene set precision (Table 2, available as supplementary data at *Bioinformatics* online). Thus, iLR is not biased toward the majority class as long as there is sufficient representation of the minority cell group.

To evaluate the impact of different choices of regularizer and regularization strength, we tested L1 and L2 regularization terms in the logistic regression model, each with regularization strengths $C\in [10^{-5},1]$. We defined an F1-like score to jointly consider classification accuracy and gene set precision in model evaluation across eight simulated datasets with different DE scale factors (Fig. 6A, available as supplementary data at *Bioinformatics* online) and different cell proportions per group (Fig. 6B, available as supplementary data at *Bioinformatics* online). Overall, L2 outperformed L1. This is expected given the iterative removal of genes in iLR, which relies on coefficient magnitudes. The shrinkage induced by L1 thus provides insufficient information for iterative gene removal. Comparing different regularization strengths for L2, we observed that a stronger regularization strength (i.e. smaller *C* values) led to worse performance for imbalanced datasets. Hence, the default value of *C* is set as C=0.1, and we recommend users explore C values ranging C∈[0.001,0.1].

### 3.2 Validation of iLR on snRNA-seq of ASD patients

**Figure 3:**
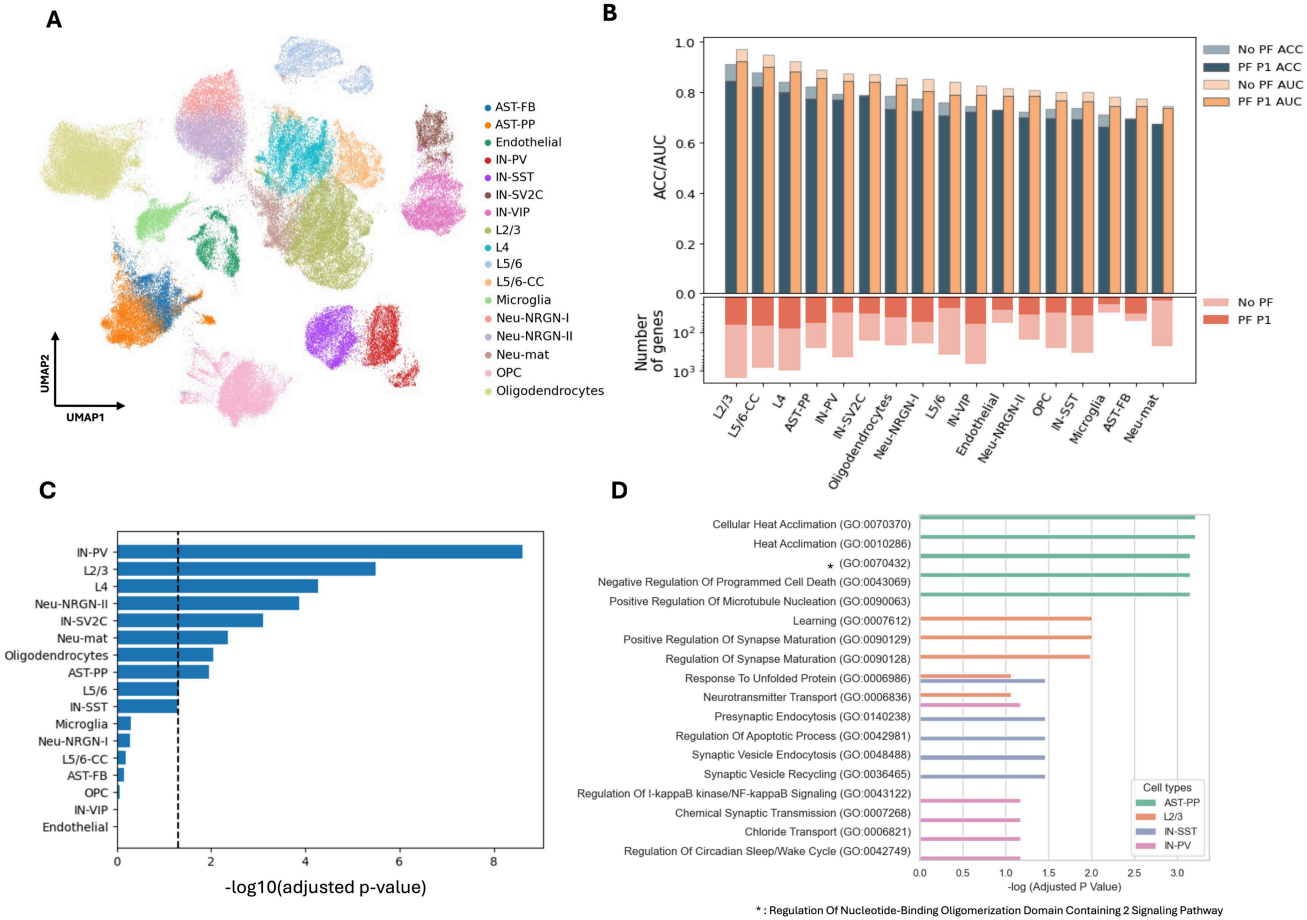
Assessment of iLR on neuronal cell fates in the brain. (A) UMAP depicting the 17 cell states identified in healthy control and ASD in data from Velmeshev *et al*. (2019). (B) iLR classification test accuracy (on ASD versus control) and gene set size for each neuronal cell state. Comparison without (bold) versus with (pale) a Pareto front of penalty 1 applied. (C) Enrichment in SFARI of the genes identified by iLR for each neuronal cell state. The dashed line denotes an adjusted *P*-value of .05. (D) Gene set enrichment analysis for genes identified in AST-PP, L2/3, IN-SST, and IN-PV cells.

To evaluate the performance of iLR to distinguish biological differences in human cell types, iLR was applied to a total of 17 cell states in the human brain to compare healthy versus ASD differences (Velmeshev *et al.* 2019) (Fig. 3A). Three different criteria were used to select the optimal gene set: the gene set with the highest AUC (i.e. no Pareto front applied), the optimal gene set according to the Pareto front without penalty, and the optimal gene set according to the Pareto front with penalty 1 (Fig. 3B, Fig. 2A, available as supplementary data at *Bioinformatics* online). Similar to the results obtained on simulated data (Fig. 2B, available as supplementary data at *Bioinformatics* online), applying a Pareto front with penalty 1 produced smaller gene sets while maintaining high accuracy (AUC values) (Fig. 3B). As was observed in the analysis of synthetic datasets, we also observed here that across iLR iterations, in several cases, smaller gene sets reduced overfitting (Fig. 10, available as supplementary data at *Bioinformatics* online). Among all cell states, L2/3, L5/6-CC, and L4 exhibited the highest AUC, indicating that these cell states exhibited the clearest differences between ASD patients and controls. This agrees with previous work on the relevance of L2/3, L5/6-CC, and IN-PV to ASD via high correlations between individual-level gene expression changes and ASD clinical severity scores (Velmeshev *et al.* 2019). In Velmeshev *et al.* (2019), microglia were predicted to have many changes in terms of gene expression from ASD to healthy controls. Here, iLR did not identify many genes associated with disease status nor classify the microglia accurately with a small gene set (Fig. 3B). To investigate this, a Wilcoxon rank-sum test was performed on the training samples from each patient and all control samples. The number of significant DE genes in microglia between ASD and controls was small, indicating that microglia changes resulting from ASD are heterogeneous and the majority of DE genes in microglia from ASD patients are unique to each patient (Fig. 2B, available as supplementary data at *Bioinformatics* online). In L2/3, in contrast, many DE genes were shared among patients (Fig. 2C, available as supplementary data at *Bioinformatics* online). This highlights the utility of iLR: in cases where classification accuracies are lower, there is likely more biological variability in the dataset worth of investigation, as is the case here with the intrinsic heterogeneity of microglia.

To assess the genes identified by iLR, we calculated enrichment scores using the SFARI database (Abrahams *et al.* 2013), a comprehensive and validated repository of autism-associated genes. Using a hypergeometric test, we analyzed the enrichment of iLR-selected gene sets in SFARI for each cell state. The enrichment analysis revealed that IN-PV, L2/3, and L4 had the highest overlap with SFARI genes (Fig. 3C), consistent with findings by Velmeshev *et al.* (2019) and Wamsley *et al.* (2024). These results strongly support the conclusion that iLR can identify small sets of genes with high relevance, in this case, revealing differences between neuronal cell states in ASD vs control brains.

We conducted a GO enrichment analysis for AST-PP, L2/3, IN-SST, and IN-PV. GO analysis revealed top terms related to brain and synaptic functions (Fig. 3D). For IN-SST cells, synaptic regulation emerged as the most significantly altered term, consistent with Wamsley *et al.* (2024). Terms associated with synaptic regulation, maturation, and neurotransmitter regulation were significantly enriched in L2/3, in agreement with Wamsley *et al.* (2024); similar agreement was seen for AST-PP, enriching for apoptosis and cytoskeleton development.

These findings collectively demonstrate the robustness and biological relevance of iLR for identifying gene sets in the context of ASD that can provide valuable insights into underlying molecular mechanisms.

To study networks mediating ASD phenotypes, we constructed TF-target gene networks in L2/3 neurons, a cell type that exhibited both high classification accuracy and significant SFARI gene enrichment (Fig. 3B and C). The network predicted from the 20 iLR genes (at penalty 2) contained 28 TFs, in stark contrast to the network predicted from the 5989 genes that were DE. In the iLR network, the top five most highly connected TFs were relatively stable between control and ASD patients; however, other TFs/target genes had large gains or losses in connectivity. Notably, MEG3 was the target gene that gained the most new TF regulators in the ASD network (versus control) (Table 5, available as supplementary data at *Bioinformatics* online). MEG3 has been previously linked to autism and other neuronal disorders (Tang *et al.* 2017, Taheri *et al.* 2021, Liu *et al.* 2022, Baazaoui *et al.* 2025). DLX1, one of the MEG3-gained TF with top strength, has been found to be related to autism (Liu *et al.* 2009) and brain development (Lindtner *et al.* 2019, Rubenstein *et al.* 2024). Moreover, TP53 (and its protein form, P53), another MEG3-gained TF, has also been implicated in autism (Rastegari *et al.* 2023) (Table 5, available as supplementary data at *Bioinformatics* online). A direct regulatory relationship between TP53 and MEG3 has been documented in patients with Huntington’s disease (Wong *et al.* 2016, Baazaoui *et al.* 2025), making this interaction a highly probable candidate for involvement in autism. This specific TP53-MEG3 interaction highlights the utility of our method. In the large network built from all 5989 DE genes, this regulation (which also gained connections in ASD) would likely be overlooked. It was obscured by the sheer volume of network changes, and MEG3 itself ranked lowly among the DE genes. For fairness of comparison, we also constructed a network with fewer DE genes: the network predicted from the top 500 DE genes contained 7111 TFs, yet even among a network this size, the MEG3–TP53 interaction was not present (Tables 3 and 4, available as supplementary data at *Bioinformatics* online).

### 3.3 iLR is robust to data resampling and multicollinearity

To evaluate the stability and robustness of iLR, we performed bootstrap and ablation analyses using gene sets constructed from the ASD dataset. Across all cell types, the coefficient ranks of genes from 200 bootstrap iterations demonstrated high stability (Figs 7A, C, E, 8A, C, E, and 9A, C, E, available as supplementary data at *Bioinformatics* online). That is, genes ranking highly in the original model maintained a consistently high rank across the bootstrap samples, ensuring they were retained in subsequent iterations. These results suggest that iLR is stable to data resampling.

To understand how multicollinearity impacts iLR results, we conducted ablation analysis. This involves systematically removing each gene from a gene set one by one, thus breaking any collinear relationships between the removed gene and the other genes comprising the gene set. This is a way in which we can also investigate and quantify the importance of each feature. For each one-gene-removed gene set, we calculated the drop-in-AUC for logistic regression using this set versus the full model, and then calculated the Spearman correlation between the drop-in-AUC coefficient ranks and the original coefficient ranks. In several cell types, including IN-PV, L5/6, and L2/3, this correlation was highly significant (Figs. S7B, D, F, S8B, D, F, and S9B, D, F). A meta-analysis across all cell types confirmed a significant positive correlation, with a 95% confidence interval of (0.27–0.52). These results show that even when faced with multicollinearity between genes, iLR still reliably identifies and prioritizes important features.

### 3.4 iLR genes capture treatment effects of immunotherapy in cell types that translate across TMEs

**Figure 4:**
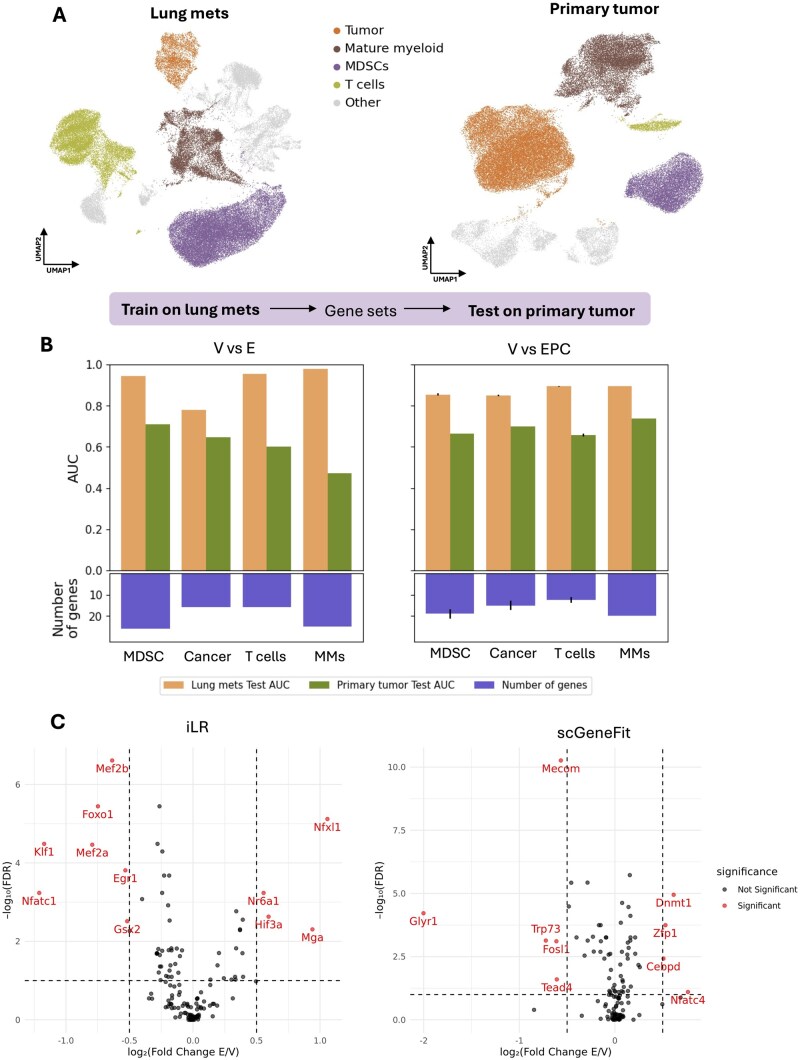
Analysis and discovery of the effects of immunotherapy on MDSCs. (A) Illustration of the high-level cell states that are shared between TME in the primary breast and the metastatic lung. (B) The accuracy of iLR in classification (via test AUC) for models trained on the lung mets and tested on the primary tumor. (C) Results of GRN inference by SCORPION. Differential activity of Inhibitory TFs based on networks inferred using gene sets marking for the change with E or with EPC with respect to V, using iLR (left) or scGeneFit (right).

We next applied iLR to two large scRNA-seq datasets describing cell types present in different tumor microenvironments (TMEs), where tumors are treated with immune checkpoint inhibitors combined with entinostat, a histone deacetylase inhibitor (Sidiropoulos *et al.* 2022, Baugh *et al.* 2024). With limited prior knowledge of the effects of treatment across cell types from different TMEs, we applied iLR to compare genes marking for treatment differences between the primary tumor (breast TME) and at metastatic sites in the lungs (lung TME). Using iLR, we analyzed MDSCs, T cells, mature myeloid cells, and the tumor across these TMEs (Fig. 4A).

We first identified cell type-specific gene sets marking for treatment effects in the lung TME, and tested these for their ability to train a classifier for the same cell type in the breast.

For changes induced by EPC treatment, all four cell states showed generally consistent AUCs tested on the primary breast TME (Fig. 4B). This suggests that these gene sets identified based on lung metastatic differences can partially explain the effects of EPC in different TMEs. In contrast, comparison of V versus E treatment showed variable results, where the mature myeloid population in the breast could not be classified using the lung mets iLR gene set (Fig. 4B). We also performed the reciprocal analysis: identifying gene sets using iLR in the breast TME and testing them on cell state differences in the lung TME (Fig. 3B, available as supplementary data at *Bioinformatics* online). The results suggest a commutability in the classification of cells based on iLR gene sets across these TMEs, indicating underlying similarities in transcriptional shifts with treatment.

Notably, the gene set inferred from mature myeloid cells in the primary breast TME is more generalizable than that of the lung TME (Fig. 3B, available as supplementary data at *Bioinformatics* online). Direct comparisons of marker gene sets revealed limited overlap between TMEs (Fig. 3A, available as supplementary data at *Bioinformatics* online).

Going beyond unsupervised assessment of iLR gene sets, several biologically important genes were identified. In the mature myeloid cells from the lung TME, iLR identified two genes (out of an 18-gene set) associated with the effects of EPC that are highly relevant to cancer progression: *Vim* and *Ccr5*.

Both these genes were ranked lower than 250th place by *P*-value using a Wilcoxon rank-sum test. *Vim* is a mesenchymal marker in tumor cells, and recent studies have shown that *Vim*-high macrophages promote tumor progression in hepatocellular carcinoma (Qiu *et al.* 2024). *Ccr5* inhibition has been associated with anti-tumoral macrophage morphology (Jiao *et al.* 2019).

### 3.5 Analysis of iLR gene sets from MDSCs yields insights into mechanisms of immunosuppression

Given the known role of MDSC immune suppression in breast cancer outcomes (Christmas *et al.* 2018, Kreger *et al.* 2023), but one for which the mechanisms are uncertain/as-yet unknown, we investigated potential mechanisms of suppression through further analysis of gene sets characterizing differences in MDSCs with/without entinostat treatment. We first compared gene sets characterizing the effect of combination therapy (EPC treatment) as identified by iLR or scGeneFit against clinical data from our phase 1b trial (Roussos Torres *et al.* 2024). Among the significant DE genes with EPC found via bulk RNA-seq on patient biopsies before and after treatment, eight genes overlapped with the 35-gene iLR gene set.

Among the genes found both by iLR and in clinical RNA-seq, *Lcn2* is a well-established non-invasive diagnostic and prognostic marker for breast cancer progression (Hu *et al.* 2018). Cd52 has been reported as a prognostic marker with the potential to predict the progression and stages of breast carcinoma (Ma *et al.* 2021). Similarly, *Rps29*, a ribosomal protein gene, is known to induce apoptosis and enhance the efficacy of anti-tumoral drugs (El Khoury and Nasr 2021). In contrast, analysis of the comparable gene set identified using scGeneFit found some overlap (*Cd52*), but overall, fewer genes were relevant to tumor progression. Additionally, the scGeneFit gene set contained a higher proportion of ribosomal genes, which are considerably less biologically informative. These findings suggest that iLR-identified genes from scRNA-seq in mouse models can provide valuable insights into disease progression with translational relevance.

To further investigate the gene sets characterizing treatment effects of EPC in MDSCs, we studied gene regulatory interactions using the gene sets inferred by iLR or scGeneFit. We inferred gene regulatory networks using SCORPION (Osorio *et al.* 2024), which uses a message-passing algorithm and is specifically designed for the comparison of regulatory networks across conditions. We ran SCORPION using either the iLR or the scGeneFit gene set as input and studied the resulting sets of TFs, which were predicted to be significantly altered by treatment (Fig. 4C). We performed gene set enrichment analysis (GSEA) on the sets of TFs output by SCORPION, but found that there were few significantly enriched terms represented by more than one gene, and those that were represented by multiple genes mostly related to unspecific molecular processes. Of note, although not significant, “myeloid cell differentiation” was identified in the GSEA of iLR-predicted TFs, represented by one gene, *Klf1*.

Further analysis of the network results via reference to the literature identified several TFs predicted from the iLR gene set that were involved in myeloid cell differentiation or suppressive functions of MDSCs. *Egr1* restricts differentiation of myeloid cells to the macrophage lineage (likely to be more suppressive) over the granulocyte lineage (Nguyen *et al.* 1993). *Egr1* also induces matrix metalloprotease 9 (*MMP9*) expression in MDSCs, which can increase invasion in the TME (Aliper *et al.* 2014). *Foxo1* has been found to promote MDSC differentiation toward more mature myeloid cells; *Foxo1* deficiency can decrease MDSC suppressive function (Tan *et al.* 2022, 2025). Other TFs corroborate these predictions: *Nfatc1* shares targets with *Foxo1* and *Mef2a/b* in directing myeloid cell differentiation to affect MDSC fates.

Analysis of the TFs predicted by SCORPION using scGeneFit genes as input did not find direct evidence linking TFs to the regulation of myeloid cell fates. While *Mecom, Fosl1*, and *Trp73* are all well-established in acute myeloid leukemia, they exert contrasting functional roles: *Mecom* is key for stem cell maintenance, whereas the latter two drive active proliferation and cell cycling (Nishida *et al.* 2018, Yang *et al.* 2023, Pastoors *et al.* 2025). *Tead4*, a well-known TF involved in embryonic development, is involved in myocyte rather than myeloid differentiation (Benhaddou *et al.* 2012). The presence of epigenetic regulators (*Dnmt1, Glyr1, Cepbd*) may in part be due to the effects on MDSCs of the epigenetic regulator entinostat.

## 4 Discussion

We developed iLR to identify small and informative gene sets that explain treatment effects or disease conditions from scRNA-seq data through the accurate classification of single cells. Key to the success of iLR is the use of Pareto front optimization to balance the trade-off between gene set size and accurate cell classification. We validated iLR on simulated datasets and on clinical data classifying ASD patients. We tested iLR on its ability to characterize the effects of immunotherapy across different TMEs, where we found iLR gene sets to be generalizable. Immunotherapy-associated genes identified by iLR included *Lcn2* and *Cd52*. We also demonstrated its utility through downstream analyses via gene regulatory network inference, which can identify key regulatory changes with treatment.

The transition from high-dimensional genomic data to mechanistic understanding is often impeded by the size of the candidate gene lists; experimental validation of a majority of these is impractical. By distilling these high-dimensional signatures into compact gene sets, we can mitigate against model and at once generate experimentally testable hypotheses. Downstream analysis such as via regulatory networks, can also benefit from smaller sets of input genes, which we found were able to identify crucial interactions (e.g. in the case of *MEG3*—*TP53*). iLR provides a flexible framework to generate gene sets of different sizes to meet the demands of various downstream analyses. For example, when performing gene set enrichment, users may choose to relax the iLR penalty to increase the size of the resulting gene set.

iLR gene sets highlighted biological mechanisms of interest. Nonetheless, any gene set predicted via linear models is limited in its scope: downstream & nonlinear analyses of genes inferred by iLR or otherwise are important. These might include cell-cell communication network inference (Jin *et al.* 2021, Mitchell *et al.* 2025), GRN inference (Kamimoto *et al.* 2023, Wang *et al.* 2023, Rommelfanger *et al.* 2025), or generative modeling (Lopez *et al.* 2018, Mitra and MacLean, 2021). TFs predicted through GRN inference that target adhesive or secreted proteins offer potential to bridge from cell-internal signaling pathways to extracellular signaling, as other methods have employed (Yan *et al.* 2025).

iLR-identified genes highlighted biologically relevant mechanisms in the cases of both the ASD and breast cancer analyses. For ASD, the genes highlighted by iLR are closely aligned with the recent literature. In the case of breast cancer TMEs, genes identified by iLR prompted new hypotheses regarding mechanisms of immune suppression (via myeloid differentiation) and offered translational potential as they could mark for treatment effects across tumor contexts—from primary to metastatic site and even from mouse to human. ScGeneFit—especially when combined with Pareto front optimization as applied here—showed comparable performance at classification tasks and offers an alternative to iLR. However, in analyses of TMEs, scGeneFit identified gene sets consisting of many less biologically informative ribosomal genes.

In downstream analyses of gene sets via GRN inference, scGeneFit identified well-known regulators, whereas iLR identified putatively new regulators of immune function, potentially guiding discovery.

While iLR demonstrated strong predictive performance, it is inherently constrained by the assumption of linear relationships in logistic regression, potentially overlooking genes with nonlinear contributions. Additionally, despite the use of L2 regularization, multicollinearity among genes can hinder the model’s ability to identify truly informative features, as correlated genes can mask each other’s effects. However, we found that for features with moderate multicolinearity (VIF < 1), the model coefficients still align with the feature importance as captured through the drop-in-AUC in an ablation test. As illustrated in this work, downstream GRN analysis may help recover such missed genes by leveraging co-expression or regulatory patterns. While iLR handles class imbalance effectively with adequate data, we saw that small sample sizes impact accurate feature recovery, although even in these cases, the models maintained high classification AUC. Finally, like other computational approaches, iLR identifies associations rather than causality; experimental validation remains essential to confirm the functional relevance of selected genes.

Overall, iLR identified small, informative gene sets that captured subtle transcriptional cell state shifts, as can often be observed with developmental disorders, cancer treatment, or other therapeutic interventions. Analysis via both *in silico* datasets and real-world scRNA-seq data demonstrated that iLR can provide interpretable signatures while maintaining high classification accuracy. Coupled with downstream gene regulatory and other analyses, iLR can help guide the discovery of biological mechanisms and new biomarkers, and, in doing so, we can better understand the transcriptional basis of complex disease.

## Supplementary Material

btag051_Supplementary_Data

## Data Availability

All code and data associated with this study are available on GitHub at: github.com/maclean-lab/iLR and archived on Zenodo at: https://zenodo.org/records/17728797.
