# Supplementary material for: Inference of marker genes of subtle cell state changes via iLR: iterative logistic regression
Source: Bioinformatics. 2026 Feb 2;42(2):btag051. doi: 10.1093/bioinformatics/btag051 (PMC12925251; doi:10.1093/bioinformatics/btag051)
Supplement: btag051_Supplementary_Data [file btag051_supplementary_data.pdf]

Supplementary Tables and Figures for
Inference of marker genes of subtle cell state changes via iterative logistic regression

Yingtong Liu[1], Aaron G. Baugh[2], Evanthia T. Roussos Torres[2,*], & Adam L. MacLean[1,*]
[1]Department of Quantitative and Computational Biology, University of Southern California, Los Angeles, CA 90089, USA

[2]Department of Medicine, Division of Medical Oncology, Keck School of Medicine, Norris Comprehensive Cancer Center, University of Southern California, Los Angeles, CA 90033, USA
[*]Correspondence: macleana@usc.edu (A.L.M.), roussost@usc.edu (E.T.R.T.).

DE scale factor	Sample size	Number of DE genes	Number of iLR genes (penalty = 1)
0.1	500	4	16
0.1	2000	34	16
0.2	500	28	16
0.2	2000	85	20
0.25	500	42	12
0.25	2000	105	20
0.5	500	81	16
1	500	222	12

S1 Table. The number of significant differentially expressed genes (adjusted p-value cutoff 0.05) identified by Wilcoxon rank sum test in eight simulated data.

Class Ratio	Sample Size	Train mean 5-CV auc	Number Selected Genes (p = 0)	Test AUC	Gene Set Precision
**5:5**	500	0.999	64	0.940	0.797
	2000	0.991	128	0.955	0.648
**4:6**	500	0.998	51	0.954	0.745
	2000	0.989	128	0.955	0.633
**2:8**	500	0.999	51	0.895	0.706
	2000	0.994	128	0.964	0.641
**1:9**	500	0.998	32	0.920	0.656
	2000	0.992	102	0.939	0.657

S2 Table. Accuracy of iLR applied on simulated data (DE scale factor = 0.25) with imbalanced classes.

S3 Table. Top Target Genes changed regulation from iLR gene list (p = 2) in Autism

Regulated by MORE TFs in Autism

Target gene	in-degree Autism	in-degree Control	Degree change
MEG3	17	8	9
SERPINH1	22	16	6
ZNF208	12	8	4
CADM2	22	18	4
GADD45G	22	19	3
HSPB1	23	20	3

Regulated by FEWER TFs in Autism

Target gene	in-degree Autism	in-degree Control	Degree change
RGS7	12	20	-8
TDRD1	10	15	-5
CLMP	8	12	-4
MT-ND3	15	18	-3
ZDHHC11	5	8	-3
NPAS4	13	15	-2

S4 Table. Top Target Genes changed regulation from Wilcoxon DE genes ($p - val < 0.05$) in Autism

Regulated by MORE TFs in Autism

Target gene	in-degree Autism	in-degree Control	Degree change
UBE2R2	591	179	412
ACTR3B	714	333	381
BCYRN1	700	334	366
NDEL1	737	411	326
PRKCI	633	317	316
ZDHHC7	534	225	309

Regulated by FEWER TFs in Autism

Target gene	in-degree Autism	in-degree Control	Degree change
RNF212	244	714	-470
PSME2P2	234	679	-445
DHX34	344	767	-423
ASNS	360	759	-399
FTCD	383	762	-379
ADCY6	389	765	-376

S5 Table. Interaction **MEG3** newly gained (Negative indicates inhibitory)

TF	Strength
ZNF579	-0.6671789
ZBTB18	-0.4885741
DLX1	0.4384252
FOXP2	-0.4015270
PPARD	-0.3816919
TP53	-0.3816919
NME2	0.3753079
SP100	-0.3509840
SP110	-0.3509840
SP140L	-0.3509840

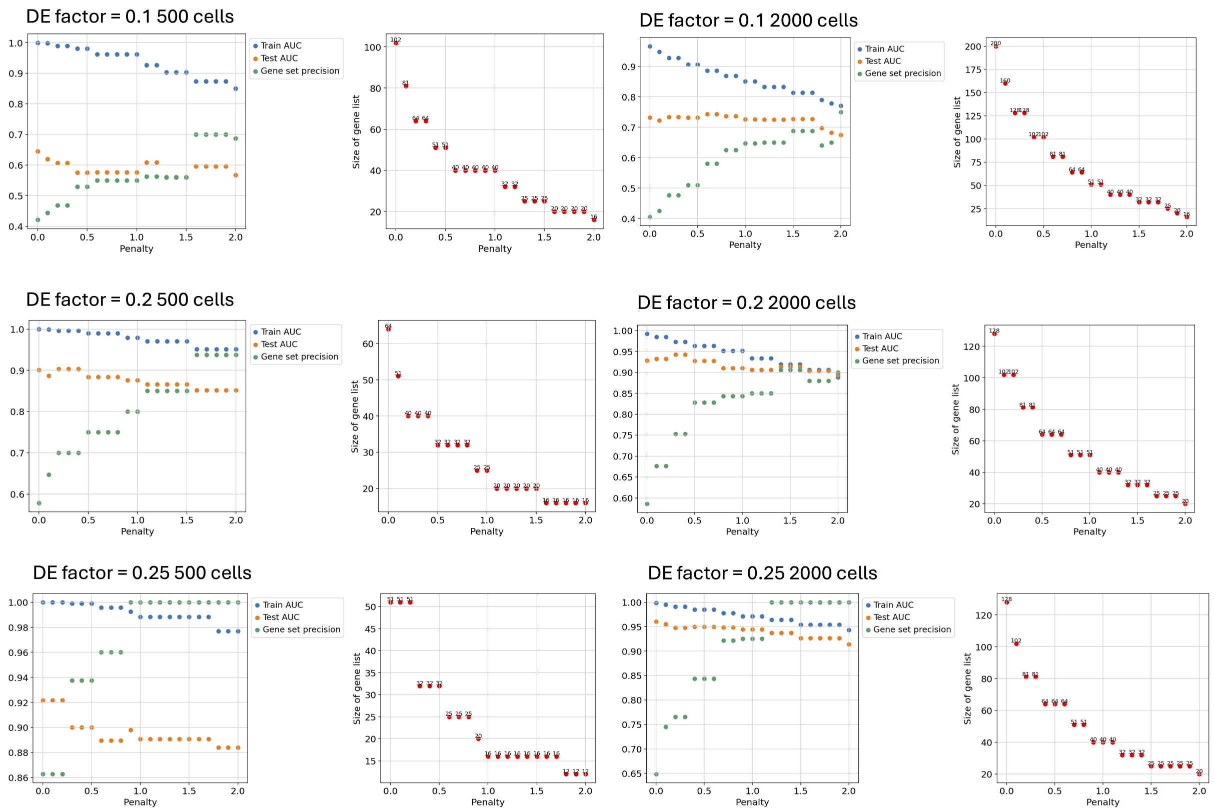

S1 Figure. iLR evaluation on simulated data. Scatter plots trace the penalty effect on four simulated datasets with different DE scale factor or sample size.

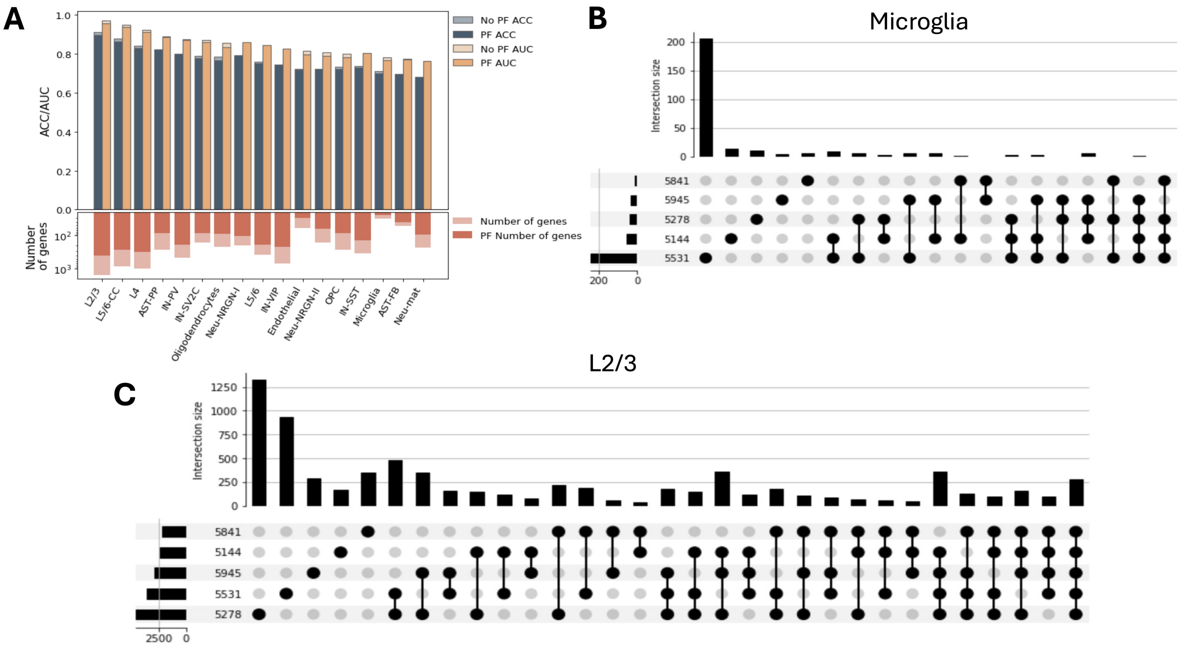

S2 Figure. iLR application on ASD dataset.(**A**) iLR classification accuracy and gene set size comparing no Pareto front (solid colored) and with Pareto front without penalty (pale colored). (**B**) Upset plot of the Wilcoxon rank sum test gene sets identified by comparison of microglia of an individual patient and all controls. (**C**) Upset plot of the Wilcoxon rank sum test gene sets identified by comparison of L2/3 of individual patients and all controls.

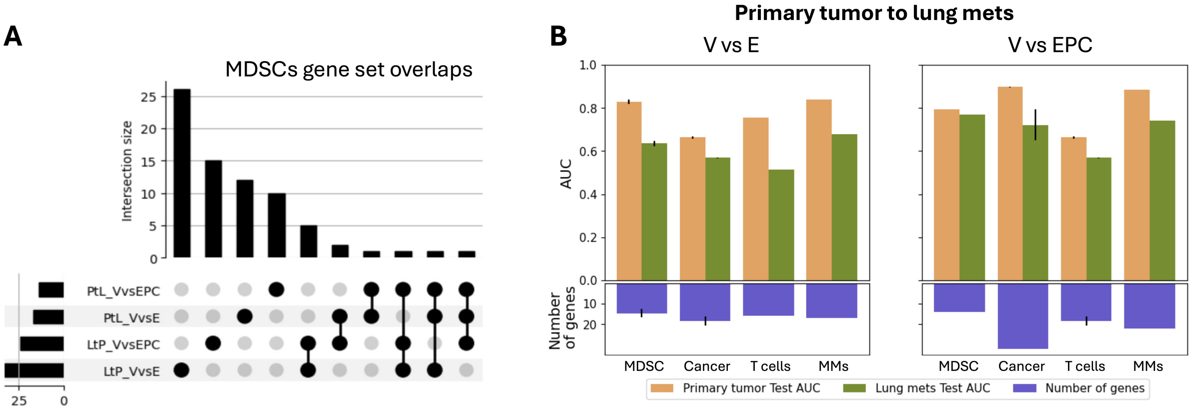

S3 Figure. Application of iLR on lung mets and primary tumor.(**A**) Upset plot showing the overlap among the iLR gene set from the four comparisons of MDSCs: lung mets V vs E, lung mets V vs EPC, primary tumor V vs E and primary tumor V vs EPC. (**B**) The test AUC for lung mets and primary tumor using the gene set drawn from the primary tumor.

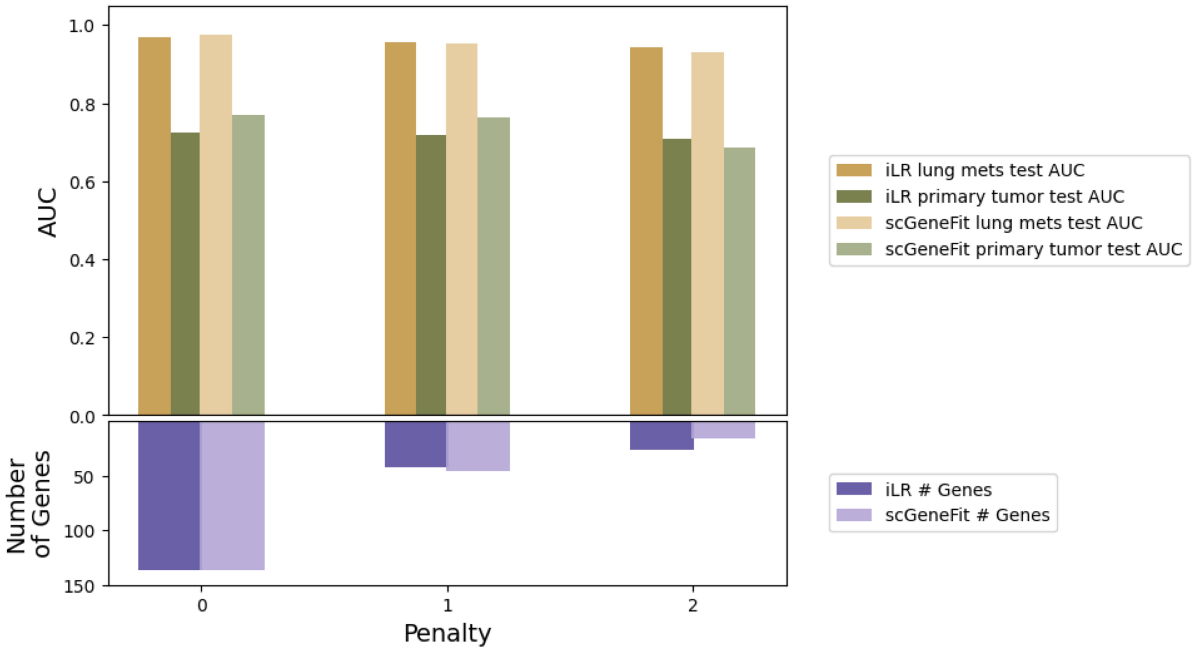

S4 Figure. Comparison of classification AUC of iLR and scGeneFit on MDSCs V vs E. Comparing the classification test AUC on lung mets and test AUC on primary tumor with the genes identified from lung mets, with the number of genes on the bottom.

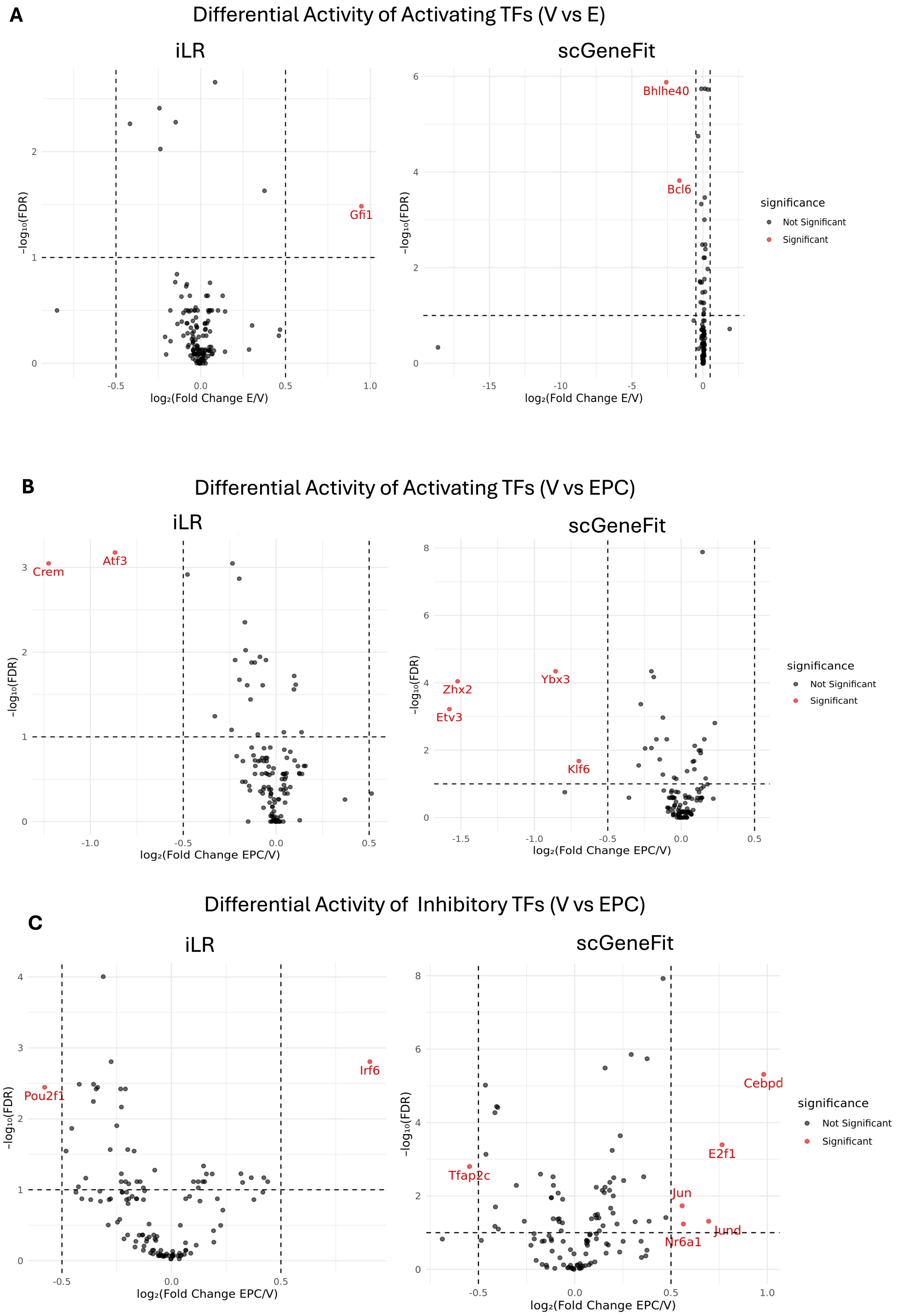

S5 Figure. TFs significantly changed inferred by SCORPION from iLR and scGeneFit genes in MDSCs (A) Significantly differential activity of activating TFs comparing V and E for iLR and scGeneFit genes respectively. (**B**)Significantly differential activity of activating TFs comparing V and EPC for iLR and scGeneFit genes respectively. (**C**)Significantly differential activity of inhibitory TFs comparing V and EPC for iLR and scGeneFit genes respectively.

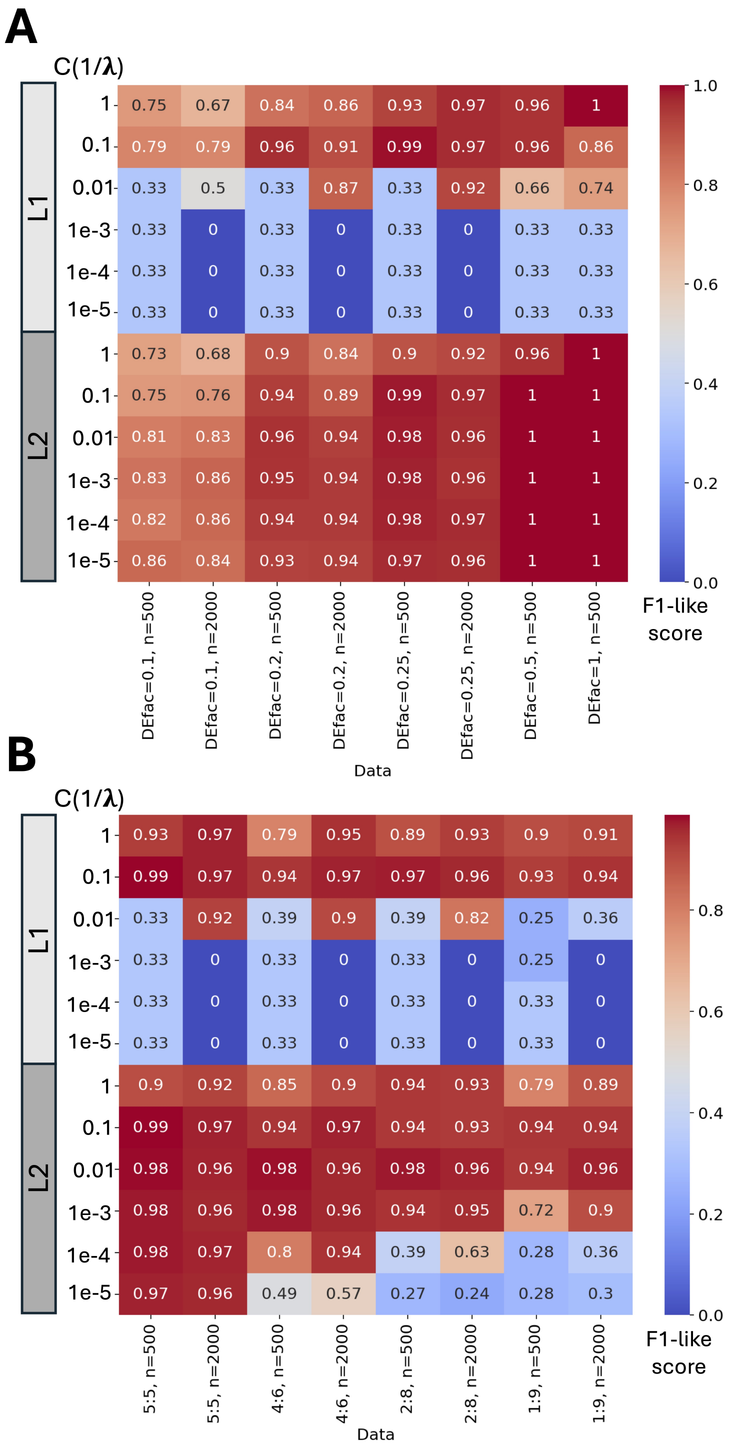

S6 Figure. Heatmap of F1-like score with different regularizer and regulation strength over simulated datasets. (**A**) F1-like score of iLR genes with penalty 0 across Splatter simulated scRNA-seq with different DE scale factor and sample size(n). (**B**) F1-like score of iLR genes with penalty 0 across Splatter simulated scRNA-seq with DE scale factor 0.25, different class proportion (5:5, 4:6, 2:8, and 1:9), and sample size(n).

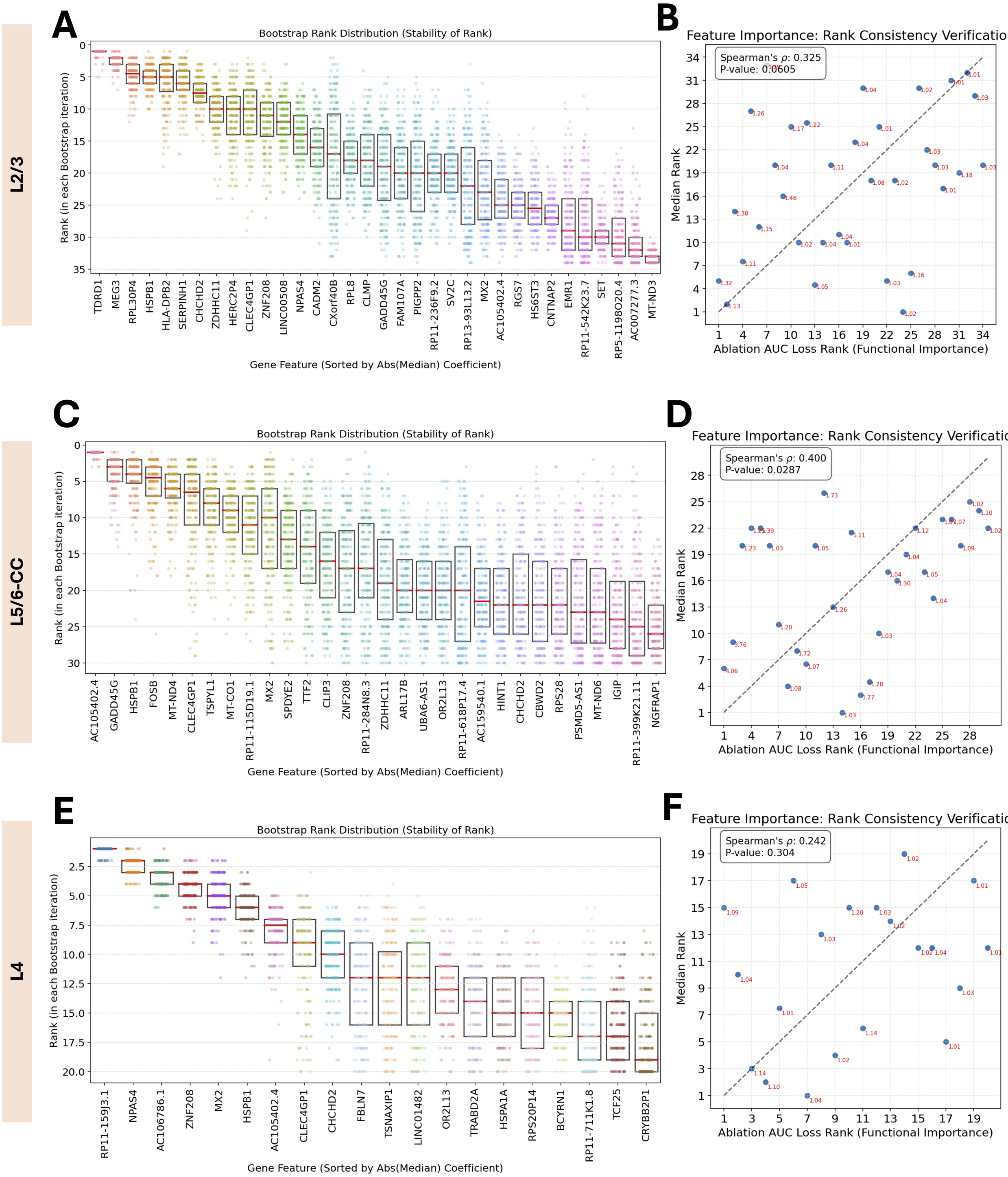

S7 Figure. Stability and ablation analysis on ASD data. (**A,C,E**) Bootstrap distribution of logistic regression coefficient ranks for iLR genes (penalty=2) in L23, L56CC, and L4 cell types (ASD data) (**B,D,F**) Spearman correlation of the bootstrap median ranks of logistic regression coefficients and AUC drop rank for each cell type.Variance inflation factors (VIF) detecting multicollinearity are shown in red beside the scatter points.

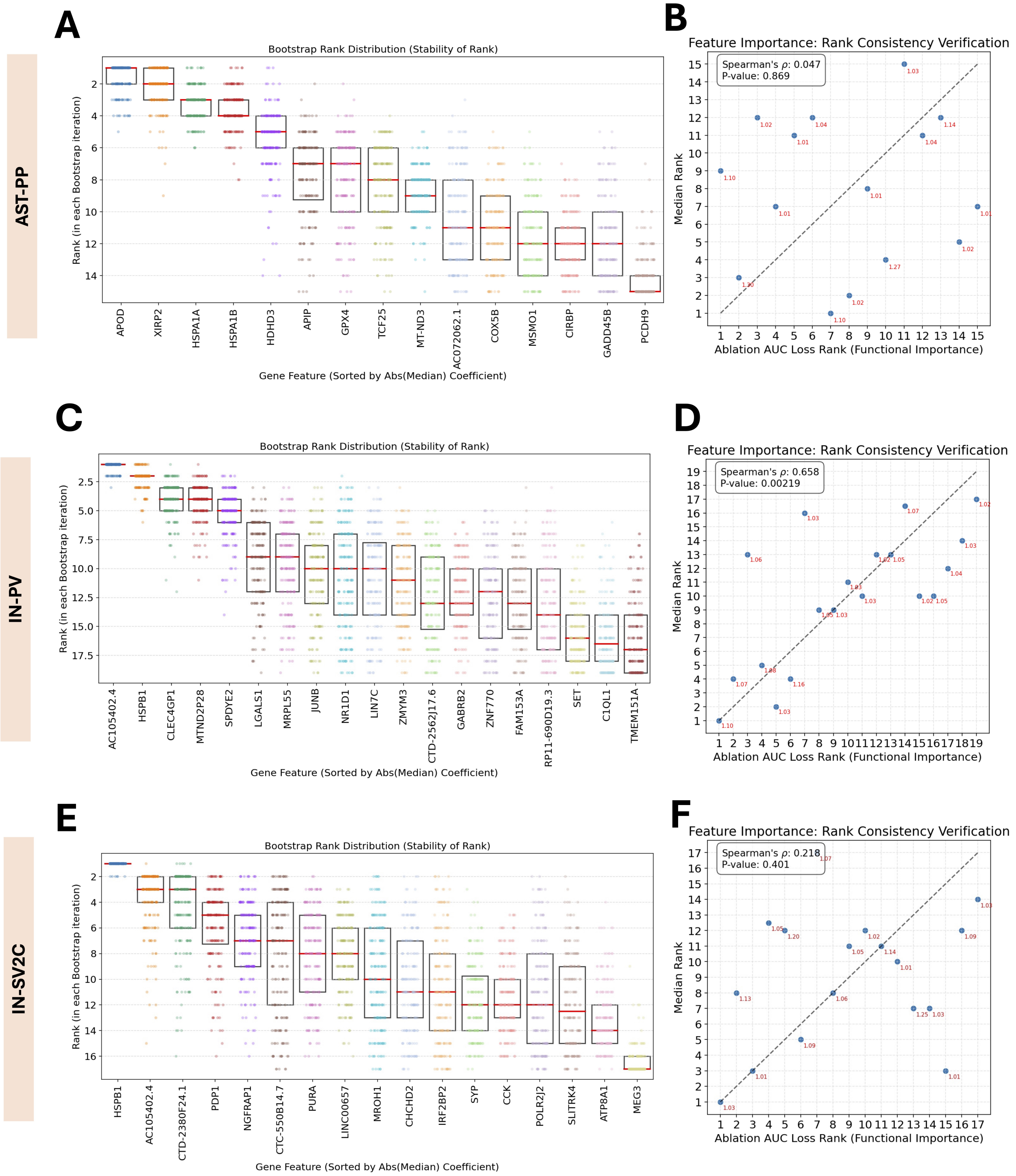

S8 Figure. Stability and ablation analysis on ASD data.(**A,C,E**) Bootstrap distribution of logistic regression coefficient ranks for iLR genes (penalty=2) in ASTPP, INPV, and INSV2C cell types (ASD data) (**B,D,F**) Spearman correlation of the bootstrap median ranks of logistic regression coefficients and AUC drop rank for each cell type. Variance inflation factors (VIF) detecting multicollinearity are shown in red beside the scatter points.

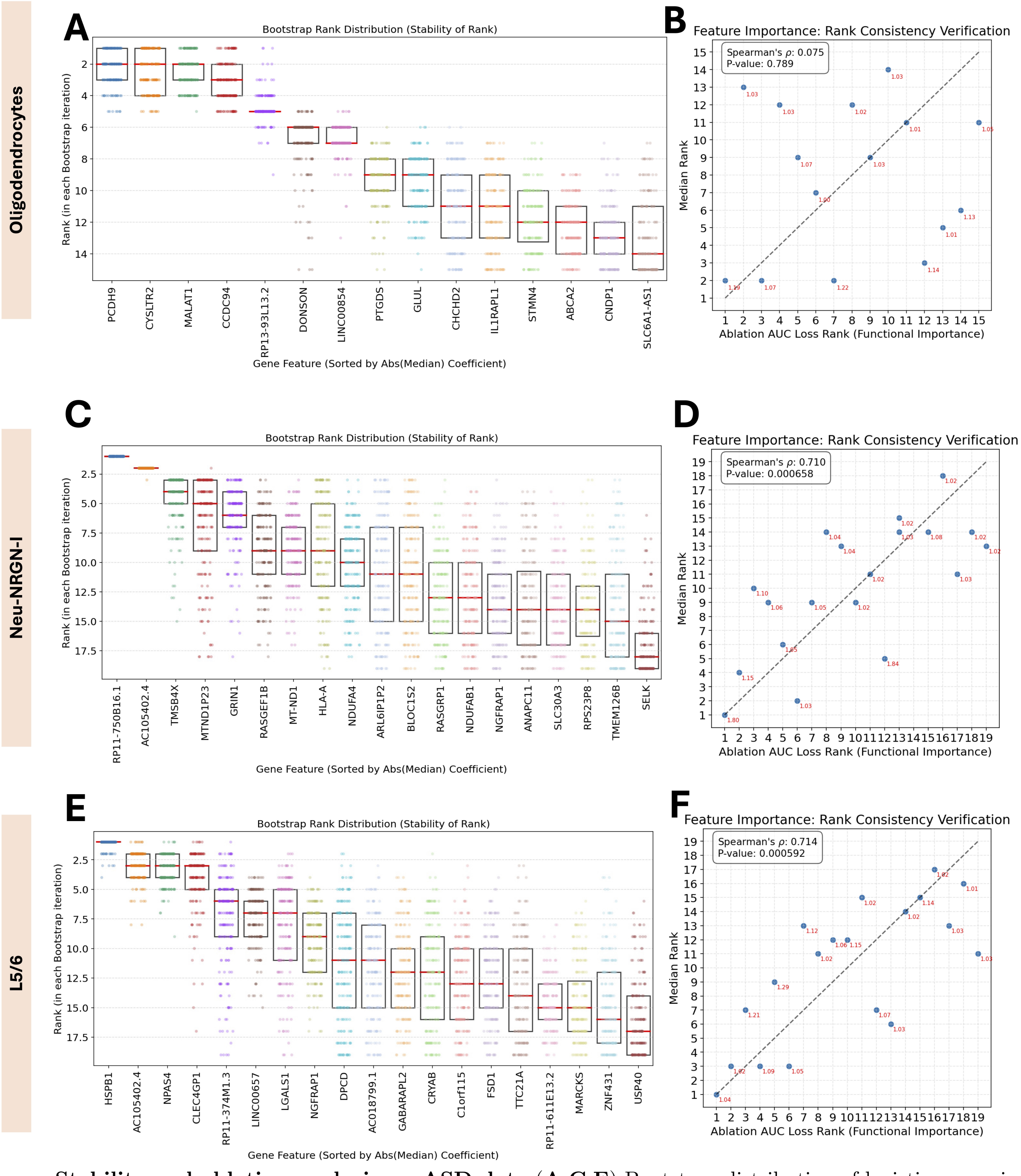

S9 Figure. Stability and ablation analysis on ASD data.(**A,C,E**) Bootstrap distribution of logistic regression coefficient ranks for iLR genes (penalty=2) in Oligodendrocytes, Neu-NRGNI and L56 cell types (ASD data) (**B,D,F**) Spearman correlation of the bootstrap median ranks of logistic regression coefficients and AUC drop rank for each cell type.Variance inflation factors (VIF) detecting multicollinearity are shown in red beside the scatter points.

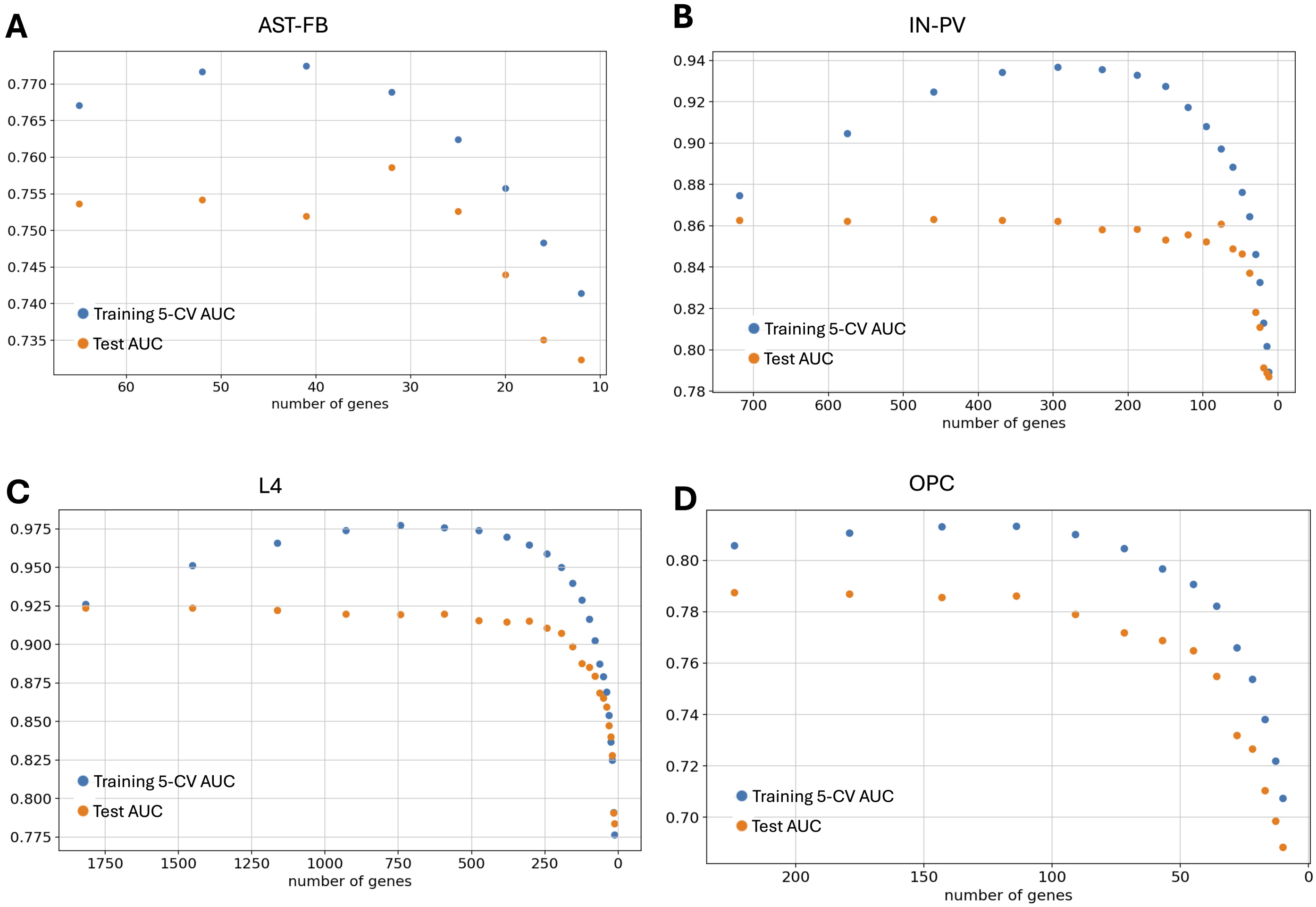

S10 Figure. Training Mean 5-CV AUC and Test AUC along iLR iteration on different cell types from the ASD dataset.
